# Sex pheromone recognition and characterization of three pheromone-binding proteins in the legume pod borer, *Maruca vitrata* Fabricius (Lepidoptera: Crambidae)

**DOI:** 10.1038/srep34484

**Published:** 2016-10-04

**Authors:** Aping Mao, Jing Zhou, Bin Mao, Ya Zheng, Yufeng Wang, Daiqin Li, Pan Wang, Kaiyu Liu, Xiaoping Wang, Hui Ai

**Affiliations:** 1 Hubei Key Laboratory of Genetic Regulation and Integrative Biology, School of Life Sciences, Central China Normal University, Wuhan 430079, China; 2 Department of Biological Sciences, National University of Singapore, 14 Science Drive 4, 117543, Singapore; 3 Key Laboratory of Insect Resource Utilization & Sustainable Pest Management of Hubei Province, College of Plant Science and Technology, Huazhong Agricultural University, Wuhan 430070, China

## Abstract

Pheromone-binding proteins (PBPs) are essential for the filtering, binding and transporting of sex pheromones across sensillum lymph to membrane-associated pheromone receptors of moths. In this study, three novel PBP genes were expressed in *Escherichia coli* to examine their involvement in the sex pheromone perception of *Maruca vitrata*. Fluorescence binding experiments indicated that MvitPBP1-3 had strong binding affinities with four sex pheromones. Moreover, molecular docking results demonstrated that six amino acid residues of three MvitPBPs were involved in the binding of the sex pheromones. These results suggested that MvitPBP1-3 might play critical roles in the perception of female sex pheromones. Additionally, the binding capacity of MvitPBP3 with the host-plant floral volatiles was high and was similar to that of MvitGOBP2. Furthermore, sequence alignment and docking analysis showed that both MvitGOBP2 and MvitPBP3 possessed an identical key binding site (arginine, R130/R140) and a similar protein pocket structure around the binding cavity. Therefore, we hypothesized that MvitPBP3 and MvitGOBP2 might have synergistic roles in binding different volatile ligands. In combination, the use of synthetic sex pheromones and floral volatiles from host-plant may be used in the exploration for more efficient monitoring and integrated management strategies for the legume pod borer in the field.

Olfactory sensation is essential for insects and vertebrates to detect air-borne chemical stimuli emitted from host plants, prey, predators, conspecifics and mates^1^,^2^,^3^. In insects, host seeking, oviposition and mating behavior are governed primarily by odor perception through sensory organs^4^. The antennae are the principal olfactory organs of insects and are highly specific sensors that exquisitely discriminate a variety of volatile chemicals that stimulate insect behavioral responses, including sex pheromones. For example, female moths emit species-specific pheromones that cause conspecific males to fly upwind to locate potential mates^5^,^6^. The sex pheromone detection system of male moths (Lepidoptera) is sensitive enough to detect a few molecules and is specific enough to differentiate between similar compounds with minor differences^7^. Because of this high specificity of insects for sex pheromones, they are effective for population monitoring and as biological control agents for the mass trapping of noxious insects in integrated pest management (IPM) programs^8^,^9^,^10^,^11^.

In Lepidoptera, pheromones and other semiochemicals are transported in the antennae by odorant-binding proteins (OBPs), which transfer the signals across the sensillum lymph to the olfactory receptors^12^. Pheromone-binding proteins (PBPs), a subfamily of odorant-binding proteins, are thought primarily to bind and transport the sex pheromones in moths (Lepidoptera)^3^,^13^. The first insect PBP was discovered in the giant silk moth, *Antheraea polyphemus* (ApolPBP), using the tritium-labeled specific pheromone (E, Z)-6, 11-hexadecadienyl acetate as a probe^13^,^14^. Since that discovery, many insect PBPs have been identified from different families within Lepidoptera^15^,^16^,^17^,^18^,^19^,^20^. For example, HarmPBP1 from cotton bollworm (*Helicoverpa armigera*) strongly binds to either of the two principal pheromone components, (Z)-11-tetradecenal and (Z)-9-hexadecenal^21^. *AipsPBP1-3* genes are highly expressed in the antennae of both male and female moths of the black cutworm (*Agrotis ipsilon*), and the binding affinities of *AipsPBP1* and *AipsPBP2* to Z7-12: Ac are much higher than those of Z8-12: Ac, whereas the binding affinity of *AipsPBP3* to Z7-12: Ac is much lower than that of Z8-12: Ac. These results indicate that the PBPs discriminate among pheromone components that have only slight structural differences^8^. Previous studies demonstrate that PBPs are small (15–17 kDa), water-soluble proteins with 6 highly conserved cysteine sequences across different species that form 3 disulfide bridges that stabilize the 3-D structure^13^,^20^,^22^,^23^. The 3-D structure of HarmPBP1 was predicted, and docking experiments indicated that the key binding site of (Z)-9-hexadecenal to HarmPBP1 included Thr112, Lys111 and Phe119, whereas the key binding site of (Z)-11-tetradecenal contained Ser9, Trp37, Phe36 and Phe119^21^. Much of the evidence demonstrates that analysis of the physiological functions of these key amino acid residues from PBPs of moths in Lepidoptera provides important cues for how different insects detect and find appropriate mates to reproduce, which has significance for increasing the efficiency of monitoring and integrated control of these pests in the field.

The bean pod borer, *Maruca vitrata* Fabricius (Lepidoptera: Crambidae), is an important tropical and subtropical pest of leguminous plants, which is widely distributed throughout Africa, Asia, South America, and the southern states of Australia^24^,^25^. The larvae feed on flowers and pods of more than 39 host plants including *Vigna unguiculat*a, which can lead to yield losses of 20–80% in sub-Saharan Africa, Southeast Asia, South Asia, and Central and South America^26^,^27^. Currently, the prevention of *M. vitrata* damage to leguminous crops primarily relies upon application of conventional chemical pesticides; however, pesticide residues in the leguminous vegetables may harm the health of the consumer. Therefore, strategies that rely on the attraction of insects to their pheromones have developed as alternative means of controlling pests, and include mating disruption and mass trapping or monitoring. Using gas chromatography linked to mass spectrometry (GC-MS) and gas chromatography coupled with electroantennography (GC-EAG)^28^,^29^, (E,E)-10,12-Hexadecadienal (E10, E12-16: Ald), (E,E)-10,12-Hexadecadien-1-ol (E10, E12-16: OH) and (E)-10-Hexadecenal (E10-16: Ald) were identified as the sex pheromone components of *M. vitrata*. These three sex pheromone components have been used successfully in field trapping experiments and for pest population monitoring of *M. vitrata*^30^,^31^. Although the use of sex pheromones for insect control is based on olfactory chemoreception, the molecular and cellular mechanisms of the perception of the female sex pheromone by *M. vitrata* male moths are unknown^32^. Moreover, based on differences observed in trapping efficiency and the possibility of polymorphism, the sex pheromone components of *M. vitrata* across different geographical regions require further study for the management of this pest.

In this study, we investigated the molecular and cellular mechanisms for the perception of female sex pheromones by male *M. vitrata.* We first cloned the full-length genes of the three PBPs of *M. vitrata* and expressed them in *Escherichia coli* to determine their involvement in sex pheromone perception. We then used real-time quantitative PCR to measure the distributions of *MvitPBP1-3* transcripts in the tissues of *M. vitrata.* Furthermore, we examined the ligand-binding abilities of the recombinant *MvitPBP1-3* proteins to the sex pheromone components of *M. vitrata* using fluorescence competitive binding assays with an N-phenyl-1-naphthylamine (1-NPN) fluorescent probe. Lastly, we performed structural modeling and molecular docking analyses to explore the binding capacities and key amino acid sites of MvitPBPs and MvitGOBPs for sex pheromone and floral volatile ligands. Our systematic studies provided further detailed evidence for the involvement of MvitPBPs in semiochemical recognition, which will promote the large-scale application of the primary sex pheromone components of *M. vitrata*.

## Materials and Methods

### Insect rearing

The legume pod borer *M. vitrata* larvae and adult moths were reared on artificial diet in the laboratory of Huazhong Agricultural University (30°28′N, 114°20′E, Wuhan City, Hubei Province, China). A laboratory colony was established and maintained at 26 ± 1 °C, 60 ± 10% RH, and 14:10 h L:D. The host cowpeas (*V. unguiculata*) were cultivated in the experimental field of Huazhong Agricultural University.

### RNA extraction, cloning and sequencing

We extracted total RNA from antennae and other tissues of male and female moths using an OMEGA E.Z.N.A TM Total RNA Kit (Omega, USA). A Prime Script first-strand cDNA synthesis kit (Invitrogen, USA) was used to synthesize the first-strand cDNA, following the manufacturer’s protocols. *MvitPBP1* (Genebank: KU517652), *MvitPBP3* (Genebank: KU517653) and *MvitPBP2* (Genebank: KU517654) genes were obtained from the National Center for Biotechnology Information (NCBI), and their open reading frames (ORF) were amplified by PCR with gene specific primers. The total PCR reaction mixture of 25 μL contained 9.5 μL of ddH_2_O, 1 μL of sample cDNA, 1 μL of forward primer (10 μM), 1 μL of reverse primer (10 μM), and 12.5 μL of rTaq mix DNA polymerase (Takara, Dalian, Liaoning, China). PCR reaction conditions were as follow: 94 °C for 3 min; 30 cycles at 94 °C for 30 s, at 55–60 °C for 30 s, and at 72 °C for 30 s; and 72 °C for 10 min. PCR products were inserted into T1 vectors (TransGen Biotech., China), following sequencing by Shanghai Sunny Biotechnology Co., Ltd.

### Sequence analysis

The cDNA sequences and deduced amino acid sequences of MvitPBP1-3 were analyzed using the online program BLAST (http://blast.ncbi.nlm.nih.gov/Blast.cgi) and the Expert Protein Analysis System (http://www.au.expasy.org/). Calculated molecular weights and predicted isoelectric points were obtained through ExPASy (http://web.expasy.org/compute_pi/). N-terminal signal peptides and most likely cleavage sites were predicted by the SignalP4.1 Server (http://www.cbs.dtu.dk/services/SignalP/). MEGA 6 and DNAMAN were used for multiple alignments and construction of the phylogenetic tree for MvitPBP1-3 with similar PBPs of other insect species.

### Expression profiles of *MvitPBPs*

Tissue expression patterns of the three PBPs were assessed by real-time PCR with cDNA templates from different tissues of male and female moths. Total RNA was prepared in triplicate using TRIzol (Omega, USA), and the genomic DNA was digested with RNA-free DNase. Six primers (PBP1YF, PBP1YR, PBP2YF, PBP2YR, PBP3YF and PBP3YR, shown in Table 1) were used to determine the relative abundance of mRNA of the three *PBP* genes, with the *actin* gene used as the reference. Real-time PCR was performed on a Bio-Rad CFX 96 real-time PCR system with SYBR Green I fluorescent dye. To check reproducibility, each real-time PCR reaction for each sample was conducted in three technical replicates and three biological replicates. Real-time PCR was conducted in 20 μL reactions that contained 10 μL of 2×TransStart Top Green qPCR SuperMix, 0.3 μL of each primer, 2 μL of sample cDNA and 7.4 μL of ddH_2_O. The cycling conditions were as follow: 95 °C for 3 min; 40 cycles at 95 °C for 10 s and at 50 °C for 30 s; and melt curve at 65 °C to 95 °C for 5 s. The data were analyzed by the 2^−ΔCt^ method, and SigmaPlot 10.0 was used to draw the histogram.

### Recombinant expression of *MvitPBPs*

The entire coding regions, without the signal peptide sequence, of *MvitPBP1*, *MvitPBP2* and *MvitPBP3* were subcloned into the NcoI/XhoI and EcoRI/XhoI sites of a PET32a (+) expression vector. BL21 (DE3) *E. coli* competent cells were transformed by heat shock and colonies were grown on LB ampicillin agar plates. A single positive clone was first identified and then grown in 6 mL of liquid LB with ampicillin overnight at 37 °C. The culture was diluted to 1:100 in fresh medium and cultured for 4 h at 37 °C until the OD value reached 0.6. IPTG was added to the culture with a final concentration of 0.3 mM, and then the culture was incubated at 30 °C for 6 h. After the incubation, the cells were collected by centrifugation (5000 rpm, 3 min) and dissolved in 1 × PBS buffer. The suspension was crushed by sonication and then separated into supernatant and sediment by centrifugation (5000 rpm, 3 min). Then, MvitPBPs were purified from the supernatant using Ni ion affinity chromatography (Thermo, USA), and enterokinase was used to remove the His-tag. The size and purity of MvitPBPs were verified by SDS-PAGE analysis, which were then stored at −80 °C.

### Fluorescence binding assays

Fluorescence binding activity was determined according to the method of Sun *et al*.^33^. Emission fluorescence spectra were recorded on a Hitachi F-4500 at 25 °C in a right angle configuration, with a 1 cm light path quartz cuvette and 5 nm slits for both excitation and emission. The protein was dissolved in 20 mM Tris-HCl buffer (pH 7.4) and ligands were added as 1 mM methanol solutions. To measure the affinity of the fluorescent ligand 1-NPN to recombinant target proteins, a 2 μM protein solution in 20 mM Tris-HCl (pH 7.4) was titrated with aliquots of 1 μM ligand in methanol to final concentrations of 2–16 μM. After each PBP or ligand was added into the reaction buffer, the mixture solution was incubated for 2 min at room temperature. The probe was excited at 337 nm and emission spectra were recorded between 380 and 450 nm. The affinities of the other ligands were measured in competitive binding assays. A solution of the protein and 1-NPN at a concentration of 2 μM was titrated with 1 mM methanol solutions of each competitor over concentration ranges of 2–24 μM, depending on the ligand (the solubilities are (EPA estimates): E10E12-16:Ald, 0.5 uM; E10-16:Ald, 0.3 uM; E10E12-16:OH, 0.17 uM; and E10-16:OH, 0.97 uM in ChemSpider). The dissociation constant for 1-NPN and the stoichiometry of binding were obtained by processing the data using Prism software. Dissociation constants of the competitors were calculated from the corresponding IC_50_ values (concentrations of ligands halving the initial fluorescence value of 1-NPN), using the equation: Ki = [IC_50_]/1 + [1-NPN]/K_1-NPN_, where [1-NPN] is the free concentration of 1-NPN and K_1-NPN_ is the dissociation constant of the coplex protein/1-NPN.

### Structural modeling and molecular docking

The presumed tertiary structures of MvitPBP1-3 and MvitGOBP2 were established using the SWISS-MODEL prediction algorithm (http://swissmodel.expasy.org/) and were displayed by PyMOL Viewer (http://www.pymol.org/). A templates search was performed on the RCSB Protein Data Bank (http://www.rcsb.org/pdb/home/home.do) using Position-Specific Iterated BLAST. After evaluating the fit between the sequences and each of the alternative 3-D models, the model with the highest score was chosen. The crystal structures of BmorGOBP2 (PDB: 2wck) and BmorPBP1 (PDB: 2p70) were used as templates to construct the three-dimensional structures of MvitPBP1-3 and MvitGOBP2. Alignment was conducted with DNAMAN and ESPript (http://espript.ibcp.fr/ESPript/ESPript/). Based on the established homology model, the docking program AutoDock Vina was used to find the potential binding mode between MvitPBP1-3 and MvitGOBP2 and the ligand. The 3-D structure of the ligand was obtained from ZINC (http://zinc.docking.org/).

## Results

### Cloning and sequence analysis of *MvitPBPs*

**Figure 1:**
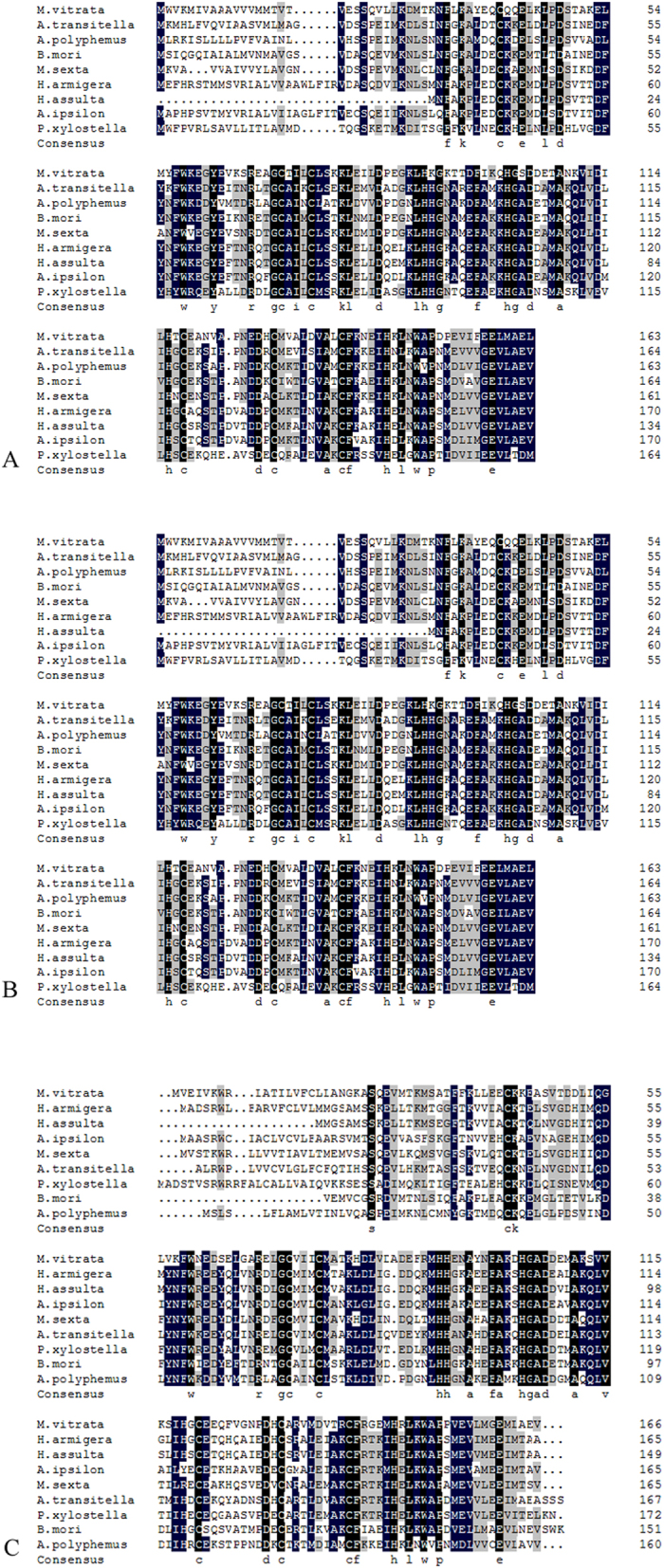
Multiple sequence alignment of MvitPBPs with other Lepidopteran insect PBPs. (**A**) MvitPBP1 is aligned with the PBP1 of other Lepidopteran moths including *Bombyx mori* (X94987.1), *Helicoverpa armigera* (HQ436362.1), *Helicoverpa assulta* (AY864775.1), *Manduca sexta* (AF117593.1), *Agrotis ipsilon* (JQ822240.1), *Antheraea polyphemus* (X17559.1), *Plutella xylostella* (FJ201994.1), *Amyelois transitella* (ACX47890.1). (**B**) MvitPBP2 is aligned with the PBP2 of other Lepidopteran moths including *Bombyx mori* (AM403100.1), *Helicoverpa armigera* (HQ436360.1), *Helicoverpa assulta* (EU316186.2), *Manduca sexta* (AF117588.1), *Agrotis ipsilon* (JQ822241.1), *Antheraea polyphemus* (AJ277266.1), *Plutella xylostella* (JX308238.1), *Amyelois transitella* (ACX47892.1). (**C**) MvitPBP3 is aligned with the PBP3 of other Lepidopteran moths including *Bombyx mori* (AM403101.1), *Helicoverpa armigera* (AF527054.1), *Helicoverpa assulta* (DQ286414.1), *Manduca sexta* (AF117580.1), *Agrotis ipsilon* (JQ822242.1), *Antheraea polyphemus* (AJ277267.1), *Plutella xylostella* (ACI28451.1).

Full-length cDNA of the three PBP genes was amplified from *M. vitrata*. *MvitPBP1-3* contained the open reading frame of 495, 501 and 519 bp, respectively. The predicted amino acid sequences of the three PBPs had the conserved six-cysteine signature that typically characterizes odorant-binding proteins (OBPs) and contained a signal peptide of 21, 24 and 30 amino acid residues at the N-terminus, respectively (Fig. 1). The calculated molecular masses of MvitPBP1-3 were 18.6, 18.0, and 19.3 kDa and the isoelectric points were 4.95, 5.24 and 5.61, respectively. Similar to other pheromone-binding proteins, SMART analysis demonstrated that MvitPBP1-3 had a single insect pheromone-binding protein domain, and their predicted tertiary structures were stabilized by three highly conserved internal disulfide bridges from four cysteine residues (positions Cys 40, Cys 71, Cys 75, Cys 118, Cys 129 and Cys 138 in MvitPBP1; Cys 42, Cys 73, Cys 77, Cys 121, Cys 132 and Cys 141 in MvitPBP2; and Cys 49, Cys 80, Cys 84, Cys 127, Cys 138 and Cys 147 in MvitPBP3; Fig. 1A–C).

### Multiple amino acid sequences alignment and phylogenetic analysis of MvitPBPs with other species of Lepidoptera

Multiple amino acid sequences alignment revealed a significant sequence similarity between the MvitPBP1-3 of *M. vitrata* and the other PBPs identified in superfamilies of Lepidoptera (Fig. 1). The amino acid sequence of the pheromone-binding protein domain of MvitPBP1 exhibited high similarity with that of CsupPBP1 from *Chilo suppressalis* (GU321120.1), BmorPBP1 from *Bombyx mori* (X94987.1) and MsexPBP1 from *Manduca sexta* (AF117593.1). The PBP domain of MvitPBP2 shared 55.05% similarity with that of AtraPBP2 from *Amyelois transitella* (ACX47892.1), 45.87% with PBP2 from *Plutella xylostella* (JX308238.1) and 44.04% with MsexPBP2 from *M. sexta* (AF117588.1). The PBP domain of MvitPBP3 was similar to that of ApolPBP3 (AJ277267.1) and AipsPBP3 (JQ822242.1), with identity values of 52.78% and 50.93%, respectively. These results demonstrated that PBPs were highly conserved among diverse species of Lepidoptera.

**Figure 2:**
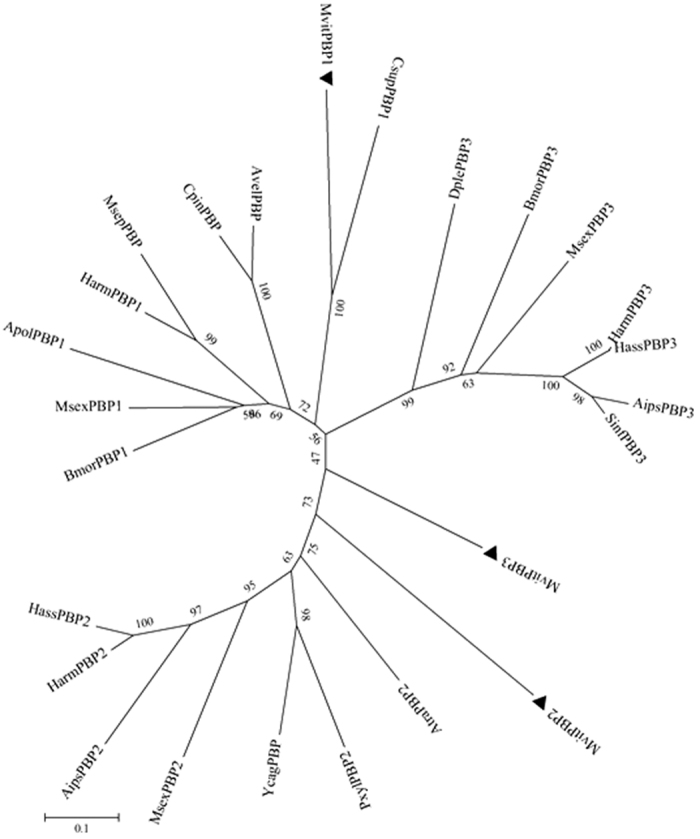
Phylogenetic tree of MvitPBPs amino acid sequence with other PBPs from different insect species. The tree was constructed by the neighbor-joining method of MEGA (v5.2). GenBank accession numbers: BmorPBP1 (X94987.1), MsexPBP1 (AF117593.1), ApolPBP2 (X17559.1), HarmPBP1 (HQ436362.1), MsepPBP (BAG71416.1), CpinPBP (AAF06135.1), AvelPBP (AAF06126.1), CsupPBP1 (GU321120.1), HarmPBP2 (HQ436360.1), HassPBP2 (EU316186.2), MsexPBP2 (AF117588.1), AipsPBP2 (JQ822241.1), YcagPBP (AAF06143.1), PxylPBP2 (JX308238.1), AtraPBP2 (ACX47892.1), SinfPBP3 (AEQ30020.1), AipsPBP3 (JQ822242.1), HarmPBP3 (AF527054.1), HassPBP3 (DQ286414.1), MsexPBP3 (AF117580.1), DplePBP3 (EHJ71308.1), BmorPBP3 (AM403101.1).

Sequences from MvitPBP1-3 and PBPs from other insects were used to construct the phylogenetic tree to assess the evolutionary relationships among the proteins. As shown in Fig. 2, MvitPBP1 was first clustered with CsupPBP1 in the phylogenetic tree, which was consistent with the highest sequence similarity between them. Then, MvitPBP2 was clustered with MsexPBP2 (AF117588.1), AipsPBP2 (JQ822241.1), HarmPBP2 (HQ436360.1), and HassPBP2 (EU316186.2), and MvitPBP3 was clustered with DplePBP3 (EHJ71308.1), BmorPBP3 (AM403101.1), MsexPBP3 (AF117580.1), HarmPBP3 (AF527054.1), HassPBP3 (DQ286414.1), AipsPBP3 (JQ822242.1) and SinfPBP3 (AEQ30020.1); thus, two large branches were formed with some of the PBP2 and PBP3 proteins from subfamilies of Lepidoptera. The three MvitPBPs were clearly separated from one another and were assigned to different subgroups, which illustrated that the three genes were highly conserved in their evolution within this family.

### Expression patterns of *MvitPBPs*

**Figure 3:**
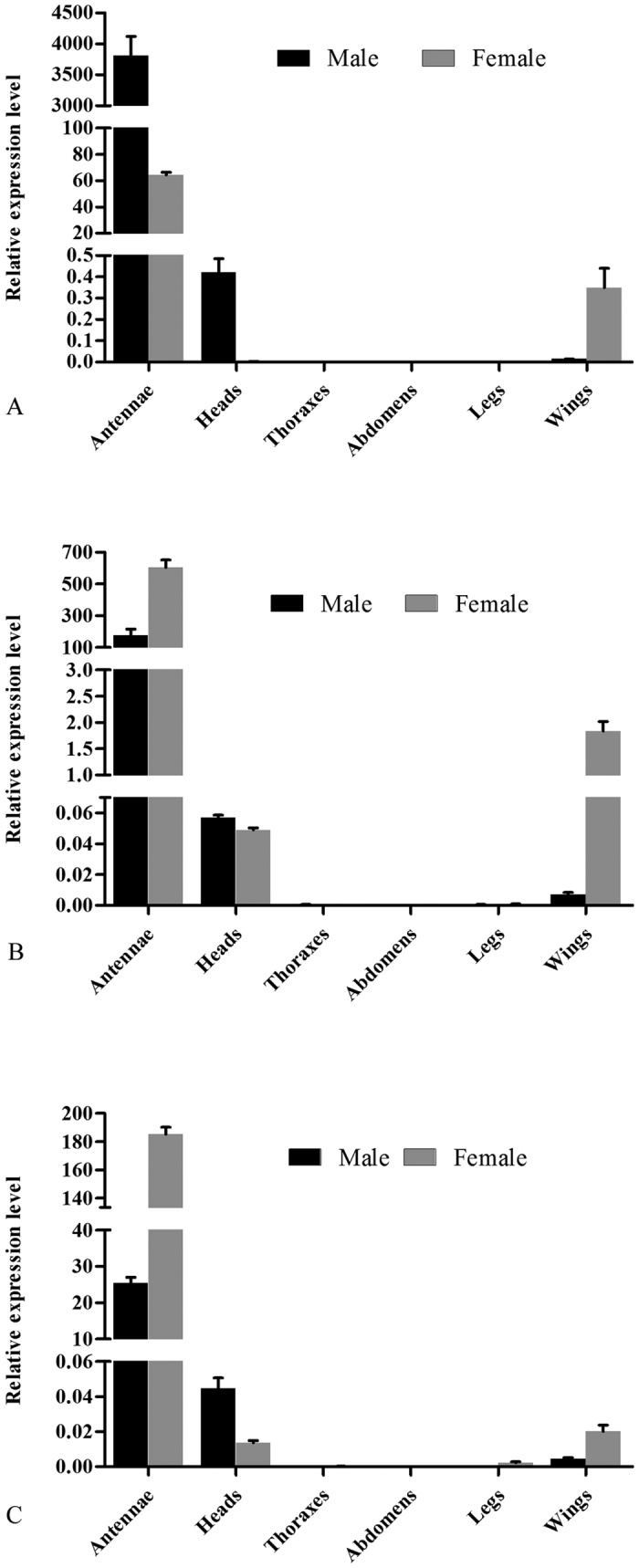
Relative transcript levels of MvitPBPs in different adult tissues and whole body measured by qRT-PCR. (**A**,**B**,**C)** are the expression level of adult tissues from MvitPBP1, MvitPBP2 and MvitPBP3, respectively.

Quantitative real-time PCR was used to measure the transcript abundance of the three *MvitPBP* genes. Transcript abundance for each PBP was determined for multiple tissues (including antenna, head, thorax, abdomen, leg and wing) from *M. vitrata*. As shown in Fig. 3, *MvitPBP1-3* transcripts were more highly expressed in antennae than in other tissues, but the expression was not specific to antennae, with low expression also detected in wing and head tissues (without antennae). Moreover, the expression of the three *MvitPBP* genes was sex-biased, and *MvitPBP1* was specifically expressed in male antennae with a 59.3-fold increase compared with that of female moths. However, compared with antennae of male *M. vitrata*, *MvitPBP2* and *MvitPBP3* were female-biased with 3.4- and 7.3-fold increases, respectively.

### Expression and purification of recombinant MvitPBPs

**Figure 4:**
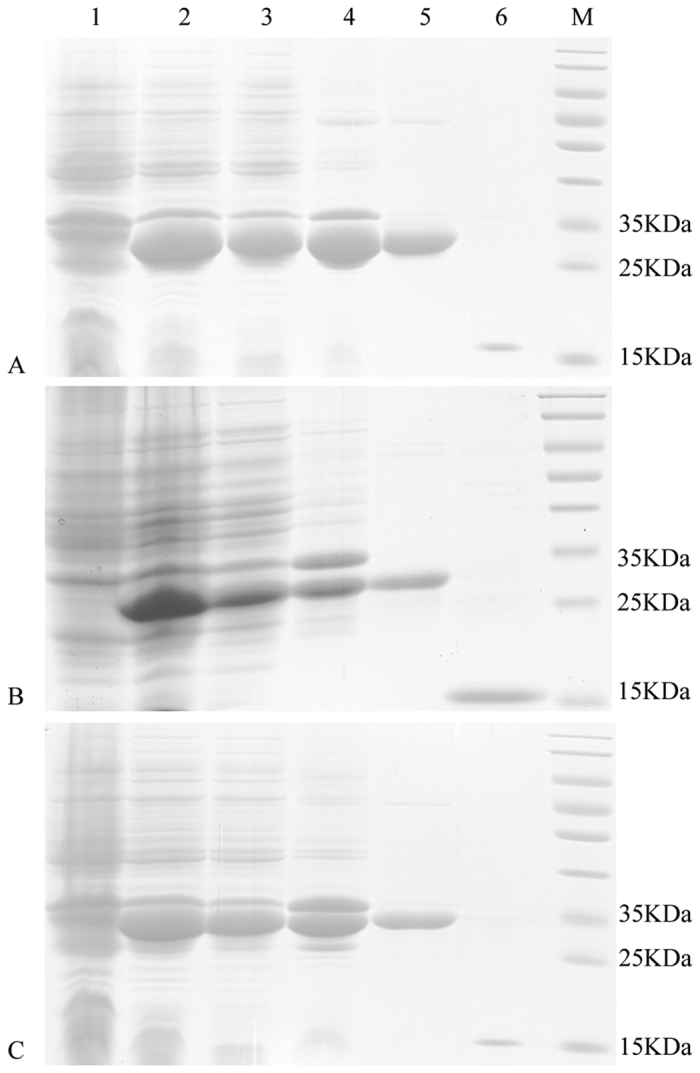
SDS-PAGE analyses of recombinant MvitPBP1 (**A**), MvitPBP2 (**B**), MvitPBP3 (**C**). Lane 1 - Non-induced *Escherichia coli* MvitPBP, Lane 2 - Induced *E. coli* MvitPBP, Lane 3 - Supernatant after broken, Lane 4 - Precipitation after broken; Lane 5 - Purified MvitPBP with His tag, Lane 6 - Purified MvitPBP without His tag, Lane M - Marker protein.

The recombinant MvitPBP1, MvitPBP2 and MvitPBP3 were abundantly expressed in *E. coli* BL21 (DE3) after IPTG induction. The three target proteins were soluble, and after expression was induced by 0.3 mM IPTG, the proteins were purified with Ni-NTA resin after ultrasonication (Fig. 4). The target proteins underwent two rounds of purification: the first round purified the recombinant protein from the total protein, and then the His-tag of the recombinant MvitPBP1-3 was removed by enterokinase. The SDS-PAGE results were consistent with the expected sizes of the MvitPBPs. Recombinant MvitPBPs were stored at −80 °C until used in the binding experiment.

### Fluorescence binding affinities

**Figure 5:**
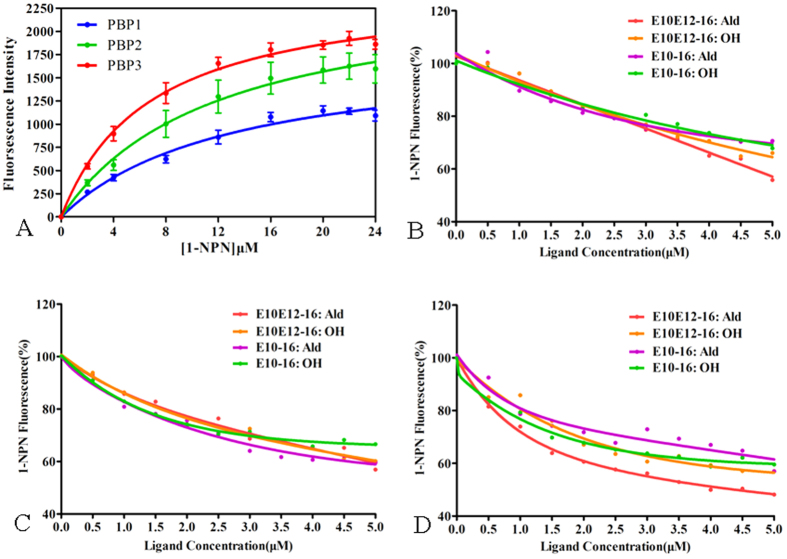
Ligand-binding experiments. (**A**) Binding curve and relative Scatchard plot. (**B–D**) Competitive binding curves of sex pheromone components to MvitPBP1-3.

The purified target proteins were used to illustrate the binding specificity of MvitPBPs using 1-NPN as the fluorescence probe in competitive binding assays, which displayed a strong blue shift in fluorescence intensity when bound to MvitPBP1-3 (Fig. S1). The binding curves and Scatchard plots indicated that the binding of the fluorescent ligand to each of the three PBPs increased with increasing concentrations of the 1-NPN (Fig. 5A). The dissociation constants of the MvitPBP1-3/1-NPN complex were 12.99, 11.86 and 6.79 μM, respectively, as calculated by Scatchard plots. The three PBPs had high binding affinities with the principal sex pheromone component E10E12-16: Ald of *M. vitrata*, and IC_50_ values (the concentration of ligand halving the initial fluorescence values) were 5.68, 5.89 and 3.67 μM, respectively (Fig. 5B–D and Table 2). MvitPBP1 and MvitPBP2 were the most sensitive to E10E12-16: Ald and E10-16: Ald, with *Ki* values (the calculated inhibition constants) of 4.97 and 6.44 for E10E12-16: Ald and 5.09 and 4.78 μM for E10-16: Ald, respectively (Fig. 5B,C and Table 2). The purified MvitPBP3 showed a strong binding affinity to E10E12-16: Ald with the *Ki* value of 2.91 μM, compared with values of 4.00, 4.82, and 4.38 μM for E10E12-16: OH, E10-16: Ald and (E)-10-Hexadecen-1-ol (E10-16: OH), respectively (Fig. 5D).

**Figure 6:**
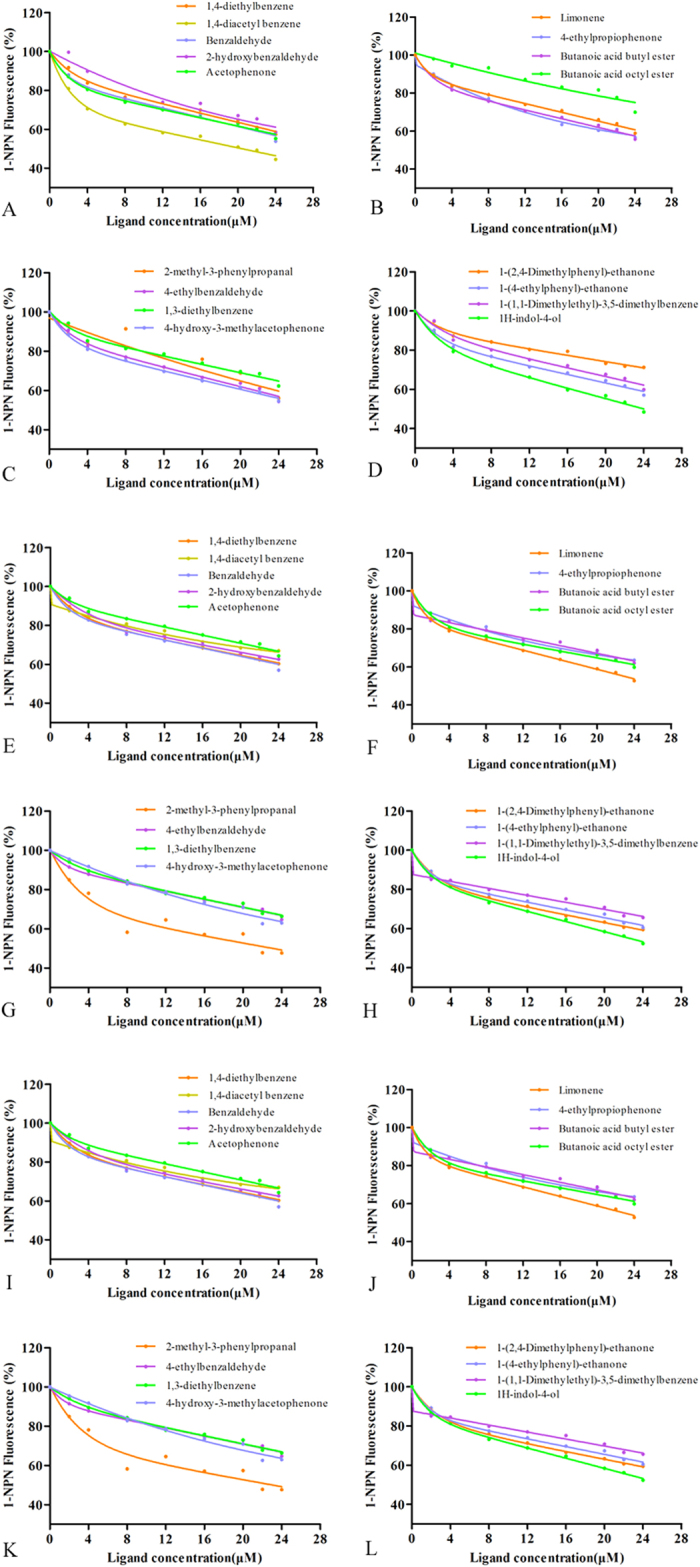
Ligand-binding experiments. (**A–L**) Competitive binding curves of seventeen host-plant volatile components to MvitPBP1-3.

Additionally, MvitPBP1-3 was tested in competitive binding assays with seventeen synthetic ligands from floral volatile chemicals that elicited obvious electroantennogram responses. Shown in Table 3 are the IC_50_ values and the calculated inhibition constants (*Ki*), when possible, for each MvitPBP/ligand combination. The binding abilities of most of the tested volatiles to MvitPBP1, MvitPBP2 and MvitPBP3 were different (Fig. 6A–L). MvitPBP3 had the highest binding affinities with butanoic acid octyl ester and 2-methyl-3-phenylpropanal of the floral volatile components, with *Ki* values of 7.64 and 8.35 μM, respectively (Table 3). Therefore, through binding with butanoic acid octyl ester and 2-methyl-3-phenylpropanal from the floral volatiles, MvitPBP3 might have a significant role in enhancing odorant signal transduction for host plant recognition in *M. vitrata*, which was similar to the role of MvitGOBP2.

### Structural modeling and molecular docking of MvitPBPs and MvitGOBPs with different ligands

**Figure 7:**
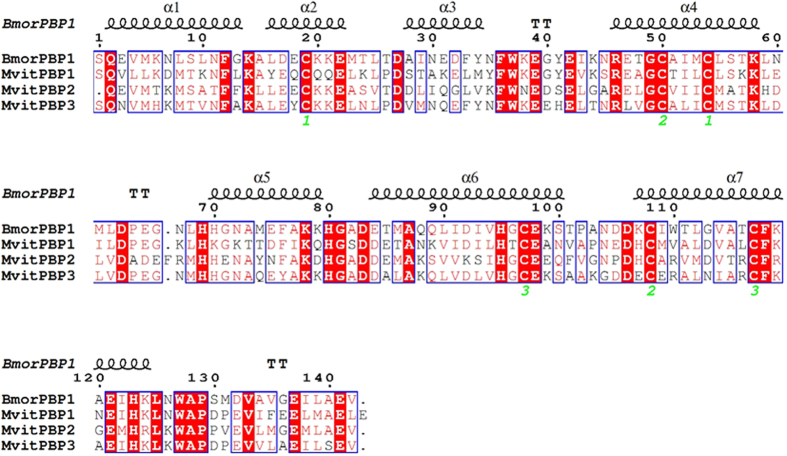
Sequence alignment of MvitPBP1-3 and BmorPBP1. Conserved residues are highlighted in white letters with a red background. Six conserved residues are labeled by pentagram. The disulfide bridges are numbered 1 to 3. α-helices are displayed as squiggles.

**Figure 8:**
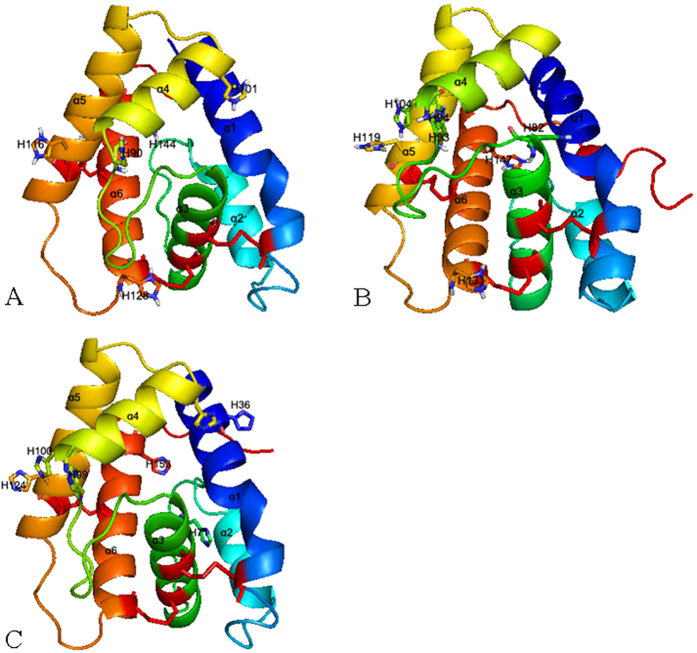
3D structural models of MvitPBP1-3. These models show some key residues and α-helices. Each monomer is colored from red to gray. Three disulfide bridges are colored in blue. His is indicated as strick.

To further investigate the binding mode and potency of MvitPBPs and MvitGOBP2 with the different tested ligands, structural modeling and molecular docking analyses were used to calculate the optimized conformation and potential key binding sites. All sex pheromone and important floral odor molecules that exhibited high binding capacities were docked into the binding cavity of MvitPBPs and MvitGOBP2. Based on the crystal structures of BmorPBP1, the structures of MvitPBP1, MvitPBP2 and MvitPBP3 were modeled. The constructed 3-D structures contained six α-helices and an additional α-helix (helix α7) (Fig. 7), which may be involved in binding and release of sex pheromones. The framework of helices was stabilized by the three disulfide bridges: for MvitPBP1, Cys40-Cys75, connecting helices α1 and α3, Cys71-Cys129, between α3 and α6, and Cys118-Cys138, between α5 and α6; for MvitPBP2, Cys42-Cys77, connecting helices α1 and α3, Cys73-Cys132, between α3 and α6, and Cys120-Cys140, between α5 and α6; and for MvitPBP3, Cys49-Cys84, connecting helices α1 and α3, Cys80-Cys138, between α3 and α6, and Cys127-Cys147, between α5 and α6 (Fig. 8). Additionally, some key residues were observed on the 3-D models of the three MvitPBPs, including a histidine (His) involved in pH-dependent conformational change^3^.

**Figure 9:**
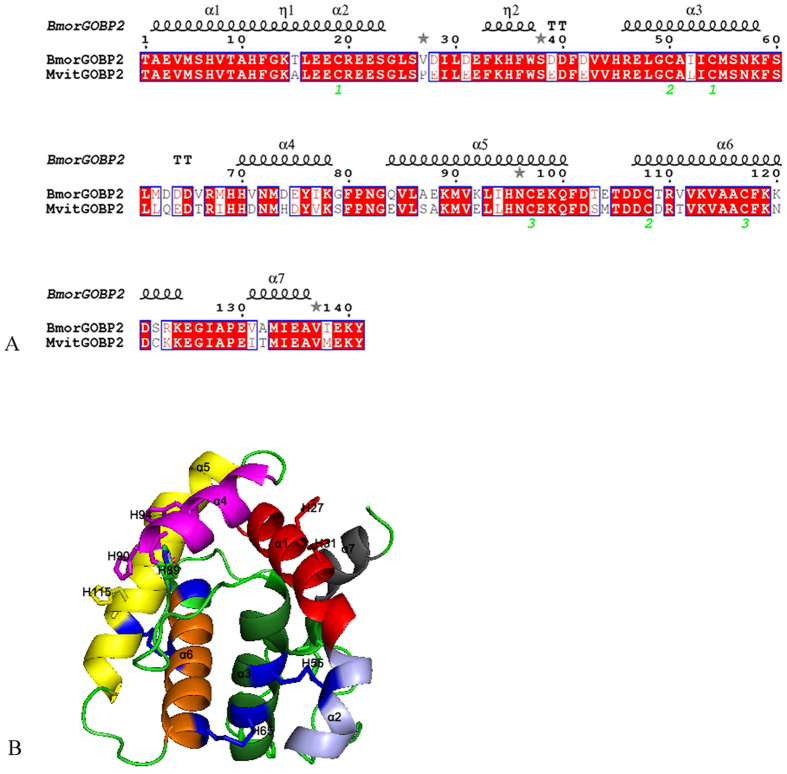
(**A**) Sequence alignment of MvitGOBP2 and BmorGOBP2. (**B**) 3D structural models of MvitGOBP2.

As shown in Fig. 9A, the amino acid sequence of MvitGOBP2 was compared with the templates of BmorGOBP2, and based on alignment analysis, the similarity of their sequences was high. Based on the crystal structures of BmorGOBP2^34^, the 3-D structure of MvitGOBP2 was also constructed by SwissModel. Some key hydrophobic and hydrophilic residues were in the amino acid sequence of MvitGOBP2, which might be involved in the binding and release of various floral volatile ligands. MvitGOBP2 was composed of six α-helices and an additional α-helix structure formed by residues 2–15 (α1a), 16–24 (α2), 46–58 (α3), 70–79 (α4), 83–101 (α5), 107–122 (α6), and 131–138 (α7) (Fig. 9B). Moreover, three disulfide bridges for stabilizing the framework of helices and the histidine (His) involved in pH-dependent conformational change are marked in the 3-D structure of MvitGOBP2 in Fig. 9B.

**Figure 10:**
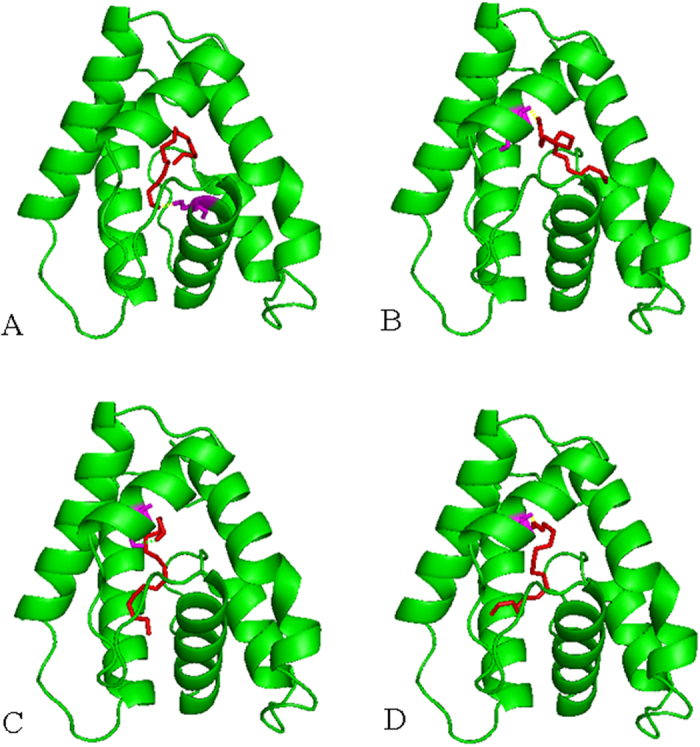
Molecular docking of MvitPBP1 and sex pheromone ligands. (**A**) E10E12-16: Ald; (**B**) E10E12-16: OH; (**C**) E10-16: Ald; (**D**) E10-16: OH.

**Figure 11:**
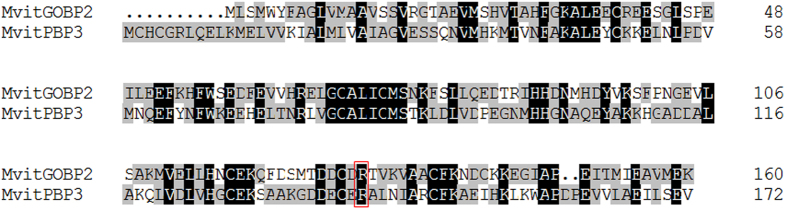
Sequence alignment of MvitPBP3 and MvitGOBP2. The letters in red box indicate the conserved residues for molecular docking.

**Figure 12:**
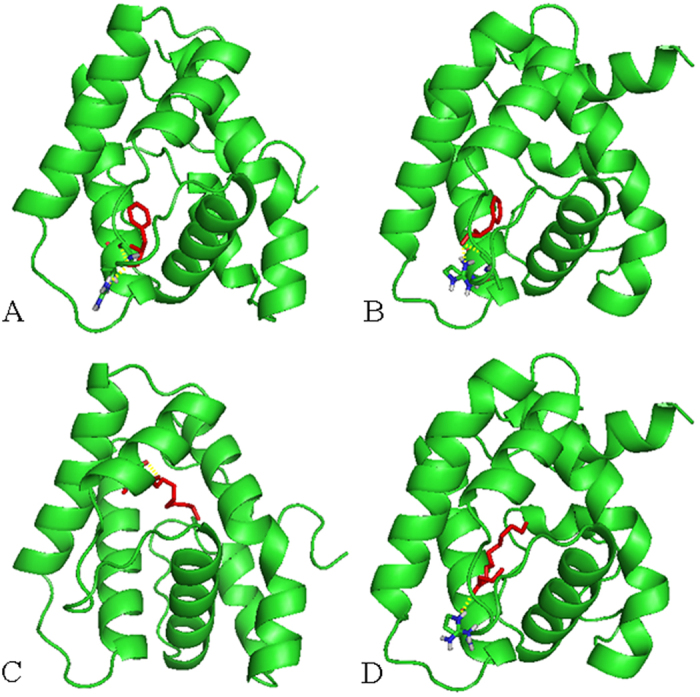
Molecular docking of MvitPBP3 and MvitGOBP2 with partial host-plant volatile ligands. (**A**) MvitPBP3 with 2-methyl-3-phenylpropanal; (**B**) MvitGOBP2 with 2-methyl-3-phenylpropanal; (**C**) MvitPBP3 with Butanoic acid octyl ester; (**D**) MvitGOBP2 with Butanoic acid octyl ester.

Additionally, based on the 3-D structural models, molecular dockings of MvitPBP1-3 and MvitGOBP2 against different ligands were performed under the identical conditions. The three PBPs had similar interactions with the sex pheromone ligands with different energy values (−5.25, −5.94, −4.95 and −5.76 in MvitPBP1; −6.13, −6.29, −5.51 and −5.57 in MvitPBP2; and −5.41, −5.64, −5.86 and −6.48 in MvitPBP3; Table 4). All the docking interaction energy values of MvitPBPs and MvitGOBP2 with ligands were negative, indicating a strong interaction between the ligand and protein. As shown in Figs 10A–D, S2A–D and S3A–D, few differences in the amino acids in the binding pocket of MvitPBP1-3 were observed. For different ligands, some amino acids appeared to be essential for ligand-binding, such as serine 77 (S77) and threonine 9 (T9) of MvitPBP1; methionine 98 (M98), glutamic acid 122 (E122), lysine 84 (L84) and glutamic acid 122 (E122)/arginine 134 (R134) of MvitPBP2; and lysine 91 (L91), methionine 32 (M32) and serine 86 (S86) of MvitPBP3. These amino acids were involved in the formation of hydrogen bonds (H-bond) between the MvitPBPs and sex pheromone ligands (Table 4). The key binding sites of MvitPBP3 and MvitGOBP2 with butanoic acid octyl ester were alanine 130 (A130) and arginine 130 (R130), respectively. As shown in Figs 11 and 12 and Table 4, MvitPBP3 and MvitGOBP2 had analogous special protein structures while sharing the identical binding sites (arginine, R140/R130) for 2-methyl-3-phenylpropanal from floral volatiles of the host plant.

## Discussion

Odorant-binding proteins (OBPs), chemosensory proteins (CSPs), chemosensory receptors (odorant receptors, ORs; ionotropic receptors, IRs), odorant-degrading enzymes (ODEs) and sensory neuron membrane proteins (SNMPs) are primarily involved in the transduction process of insect olfactory chemical signals^35^,^36^. The initial molecular interactions for chemical signals (semiochemicals) such as sex pheromones and host odors are with odorant-binding proteins (OBPs), which likely ferry the semiochemical molecules across the antennal sensillum lymph to the olfactory receptors^34^. Subsequently, the odorant molecules are rapidly degraded by ODEs, and the chemical signals are converted into electrophysiological signals to complete pheromone conduction^37^. In Lepidoptera, the odorant-binding proteins are classified into pheromone-binding proteins (PBPs) and general odorant-binding proteins (GOBPs) based on primary sequence homology^38^,^39^. Moreover, PBPs and GOBPs are highly expressed in various types of olfactory sensilla on insect antennae and play different roles in recognizing sex pheromones and volatile odorants of the host. Therefore, with the identification and functional analysis of pheromone-binding proteins in *M. vitrata* in this study, new methods can be developed for controlling this pest by interfering with olfactory perception and subsequent mating behaviors.

qRT-PCR analysis indicated that *MvitPBP1-3* genes were involved in odorant (including sex pheromones) detection because these genes were primarily expressed in the antennae of both sexes, and the level of expression was very low in other tissues such as the head, thorax, abdomen, leg and wing. *MvitPBP1* gene was more abundantly expressed in male antennae than in female antennae, and a similar pattern of expression is reported in many other lepidopterans, including *B. mori*, *Agrotis segetum*, *H. armigera*, *Heliothis virescens* and *Spodoptera exigua*^40^,^41^,^42^,^43^,^44^,^45^,^46^. Notably, the expression level of *MvitPBP2* and *MvitPBP3* genes was much lower in male antennae than that in female moth antennae, which is similar to the expression of *AipsPBP2*-*3*^8^. Moreover, based on the higher expression of *MvitPBP1* gene in male *M. vitrata* than that of the other PBP genes, *MvitPBP1* gene might have a major role in male-female recognition. Furthermore, female-biased expression of both of *MvitPBP2* and *MvitPBP3* genes might indicate involvement in the autodetection of sex pheromone compounds, which has been demonstrated in other lepidopterans^47^,^48^,^49^,^50^.

The legume pod borer *M. vitrata* is a serious pantropical insect pest of grain legumes such as cowpea (*V. unguiculata*), pigeon pea (*Cajanus cajan*) and common bean (*Phaseolus vulgaris*)^51^,^52^,^53^,^54^. Because of the economic damage caused by this pest, insecticides and sex pheromone components have been tested as different control strategies in the fields of southern China. Lu *et al*. reported that a blend of E10, E12-16: Ald; E10-16: Ald; and E10 E12-16: OH (ratio = 100:5:5) attracted significantly more males than any other bait in a field test, including the primary component alone, a two component blend or virgin females^31^. In our ligand binding experiments, the four sex pheromone components of *M. vitrata* strongly bonded with MvitPBP1-3, with different levels of sensitivity. MvitPBP1 and MvitPBP3 were the most sensitive to E10E12-16: Ald, whereas MvitPBP2 was the most sensitive to E10-16: Ald. Both of these organic aldehyde compounds are reported to be the primary sex pheromone components of *M. vitrata*^28^,^29^, and MvitPBPs showed excellent binding affinities for these two components. Moreover, the binding of E10E12-16: Ald with MvitPBP1 and MvitPBP3 was significantly stronger than that with E10-16: Ald. To explain the differences in binding capacity with MvitPBP proteins, E10, E12-16: Ald has one more unsaturated bond than E10-16: Ald, which is consistent with the binding of PxylPBPs^46^.

PBPs play major roles in sex pheromone perception by binding and transporting hydrophobic pheromone molecules across the aqueous sensillar lymph to the olfactory receptors and by discriminating different semiochemicals including plant volatiles^55^. In our previous study, 17 electroantennogram-active compounds were identified from floral volatiles of *V. unguiculata* by GC-MS and GC-EAD^25^. Based on the fluorescence binding experiments, MvitPBP1-2 had very weak ligand binding capacities with all floral volatiles from the host plant *V. unguiculata*, which were much lower than those for the sex pheromone. However, MvitPBP3 displayed higher binding capacities with partial floral volatile components than those of MvitPBP1-2. Among the 17 tested compounds, the binding activity of butanoic acid octyl ester to MvitPBP3 was the strongest and displaced half the 1-NPN from the MvitPBP3/1-NPN complex at a ligand concentration of 20 mM (Table 3), although the abundance of this ester was low and the EAG response was weaker than that of other plant volatiles^25^. Additionally, 2-methyl-3-phenylpropanal bonded strongly with MvitPBP3, which was the most abundant compound in the floral volatiles of the host plant and elicited a high electrophysiological response from antennae of *M. vitrata*^26^. MvitGOBP2 also had high binding affinities with butanoic acid octyl ester and 2-methyl-3-phenylpropanal among the floral volatile components. When the concentration of butanoic acid octyl ester and 2-methyl-3-phenylpropanal reached 8.5 and 3.81 μM, respectively, the fluorescence intensity of the MvitGOBP2/1-NPN complex rapidly decreased to approximately 50%^26^. Based on these results, MvitPBP3 and MvitGOBP2 might be derived from the same olfactory protein family because they shared similar amino acid binding sites with the identical volatile ligands.

In this study, we found that the three MvitPBPs had different energy values and key residues that interacted with ligands, with six amino acid residues of MvitPBP1-3 involved in binding a sex pheromone within a cavity. The different binding affinities of the three MvitPBPs toward the tested sex pheromone ligands was an indication of their sequence and structural differences. These results provide further support for the results from a previous field application of sex pheromones for pest population monitoring of *M.* vitrata^29^,^30^,^31^. Notably, based on the docking analysis and fluorescence binding assays, MvitPBP3 and MvitGOBP2 had similar binding energies and excellent binding capacities with the identical volatile ligand (2-methyl-3-phenylpropanal). Additionally, based on sequences alignment analysis, these two olfactory proteins had the identical amino acid residues (R140/R130) and similar protein structures around the binding cavity (Table 4, Fig. 12). These results indicated that arginine (R140/R130) might be a key binding site involved in the initial recognition of volatile ligands. Many previous studies report that PBPs and general odorant-binding proteins (GOBPs) are in different subfamilies of OBPs that are involved in the recognition and transport of pheromones and host odors through the lymph of chemosensilla^3^,^13^. Therefore, we speculate that MvitPBP3 and MvitGOBP2 may play a synergistic role in binding different types of floral volatile ligands with a high affinity, which highlights that MvitPBP3 and MvitGOBP2 are likely involved in the functional differentiation of odorant-binding protein family of *M. vitrata*.

## Conclusion

Pheromone-binding proteins are important components of insect olfactory systems, and sensitive olfaction is vital for recognition of hosts, mating and oviposition in insects^21^. In this study, we provided more evidence that MvitPBPs had excellent binding affinities with the sex pheromones and that partial floral volatiles from the host plant were key ligands of MvitPBP3. The identification and functional analysis of pheromone-binding proteins in *M. vitrata* will lead to new methods for controlling this pest by interfering with their olfactory perception. Moreover, molecular docking results for MvitPBPs and MvitGOBPs will contribute to the understanding of the multiple roles and synergistic effects of these proteins in the host seeking, oviposition and mating behavior of adult moths. Thus, synthetic sex pheromones of *M. vitrata* and two types of key floral volatiles from the host plant *V. unguiculata* may be used in the exploration for more efficient monitoring and integrated management strategies for the legume pod borer in the field.

## Additional Information

**How to cite this article**: Mao, A. *et al*. Sex pheromone recognition and characterization of three pheromone-binding proteins in the legume pod borer, *Maruca vitrata* Fabricius (Lepidoptera: Crambidae). *Sci. Rep.* **6**, 34484; doi: 10.1038/srep34484 (2016).

## Supplementary Material

Supplementary Information

## Figures and Tables

**Table 1:** Primers used in the experiments.

Primer name	Sequence (5′-3′)
PBP1-NcoF	CATGCCATGGTC CCAAGTGTTGTTGA
PBP1-XhoR	GCTCGAGCTACTCCAACTCCGCC
PBP2-NcoF	CCCATGGCAGGAGGTGATGACCAAAAT
PBP2-XhoR	CCCTCGAGTTAGACTTCAGCCAGC
PBP3-EcoRF	CATGCCATGGCT TCCCAAAATGTAATGCATAAGAT
PBP3-XhoR	CCGCTCGAG TTAGACTTCAGACAAGATCTCAG
PBP1YF	CGGCGGTGGTGGTTATGATG
PBP1YR	TAGCCGTAGAGTCGGGAAGTTT
PBP2YF	AAGGGCTGGTCAAGTTCTGGA
PBP2YR	TGTAAGCGTTCTCGTGGTGC
PBP3YF	TTGCTAAAGCTTTGGAGTATTGC
PBP3YR	GGTTCATCACGTCTGGCAGG
ActinF	AGCACGGTATCATCACCAACT
ActinR	GGTCTCAAACATGATCTGGGT

**Table 2:** The binding constants of different ligands.

No.	Compounds	MvitPBP1		MvitPBP2		MvitPBP3	
		IC_50_(μM)	*K*_i_(μM)	IC_50_(μM)	*K*_i_(μM)	IC_50_(μM)	*K*_i_(μM)
1	E10E12-16: Ald	5.68	4.97	5.89	5.09	3.67	2.91
2	E10E12-16: OH	8.84	7.73	5.91	5.11	5.05	4.00
3	E10-16: Ald	7.36	6.44	5.53	4.78	6.09	4.82
4	E10-16: OH	7.64	6.69	6.82	5.90	5.53	4.38

Binding of 1-NPN and different sex pheromone components to MvitPBP1-3. Note: IC_50_, ligand concentration displacing 50% of the fluorescence intensity of the MvitPBPs/N-phenyl-1-naphthylamine complex; *Ki*, dissociation constant.

**Table 3:** The binding constants of different ligands.

No.	Compounds	MvitPBP1		MvitPBP2		MvitPBP3	
		IC_50_(μM)	*K*_i_(μM)	IC_50_(μM)	*K*_i_(μM)	IC_50_(μM)	*K*_i_(μM)
1	Butanoic acid butyl ester	27.38	23.96	33.37	28.86	30.59	24.23
2	Limonene	30.35	26.56	24.99	21.61	26.43	20.94
3	1,3-diethylbenzene	33.97	29.73	36.16	31.27	28.94	22.93
4	1,4-diethylbenzene	28.50	24.94	29.73	25.71	25.22	19.98
5	Benzaldehyde	27.00	23.63	29.46	25.48	28.94	22.93
6	Acetophenone	27.29	23.89	36.14	31.26	38.06	30.15
7	2-hydroxybenzaldehyde	29.67	25.97	31.03	26.84	26.76	21.20
8	4-ethylbenzaldehyde	27.14	23.75	36.76	31.79	34.14	27.05
9	Butanoic acid octyl ester	46.16	40.40	30.42	26.31	9.65	7.64
10	1-(4-ethylphenyl)-ethanone	28.43	24.88	30.98	26.79	30.58	24.23
11	2-methyl-3-phenylpropanal	27.70	24.24	20.97	18.14	10.54	8.35
12	1-(2,4-Dimethylphenyl)-ethanone	41.81	36.23	28.42	24.63	32.16	27.87
13	4-ethylpropiophenone	26.55	23.24	32.20	27.85	25.11	19.89
14	4-hydroxy-3-methylacetophenone	26.26	22.98	31.78	27.48	25.76	20.41
15	1H-indol-4-ol	22.46	19.66	24.57	21.25	27.81	22.03
16	1,4-diacetyl benzene	19.63	17.18	35.64	30.82	44.63	35.36
17	1-(1,1-Dimethylethyl)-3,5-dimethylbenzene	30.99	27.12	37.16	32.14	23.78	18.84

Binding of 1-NPN and different host-plant volatile ligands to MvitPBP1-3. Note: IC_50_, ligand concentration displacing 50% of the fluorescence intensity of the MvitPBPs/N-phenyl-1-naphthylamine complex; *Ki*, dissociation constant.

**Table 4:** The docking results of MvitPBP1-3 and MvitGOBP2 with different ligands.

No.	Compounds	CDOCKER Interaction energy (Kcal/mol)				Residues forming H-bond with ligand			
		PBP1	PBP2	PBP3	GOBP2	PBP1	PBP2	PBP3	GOBP2
1	E10E12-16: Ald	−5.25	−6.13	−5.41	—	S77	M92	L91	—
2	E10E12-16: OH	−5.94	−6.29	−5.64	—	T94	E122	M32	—
3	E10-16: Ald	−4.95	−5.51	−5.86	—	T94	L84	S86	—
4	E10-16: OH	−5.76	−5.57	−6.48	—	T94	E89/R134	S86	—
5	Butanoic acid octyl ester	—	—	−4.52	−4.24	—	—	A103	R130
6	2-methyl-3-phenylpropanal	—	—	−4.76	−5.71	—	—	G96/R140	T86/R130
