# Supplementary material for: Sex pheromone recognition and characterization of three pheromone-binding proteins in the legume pod borer, Maruca vitrata Fabricius (Lepidoptera: Crambidae)
Source: Sci Rep. 2016 Oct 4;6:34484. doi: 10.1038/srep34484 (PMC5048296; doi:10.1038/srep34484)
Supplement: Supplementary Information [file srep34484-s1.pdf]

Sex pheromone recognition and characterization of three pheromone binding proteins in the legume pod borer, *Maruca vitrata* Fabricius (Lepidoptera: Crambidae)

Short title: Characterization of pheromone binding proteins from *M. vitrata*

Aping Mao [1, a], Jing Zhou [1, a], Bin Mao [1], Ya Zheng [1], Yufeng Wang [1], Daiqin Li [2], Pan Wang [3], Kaiyu Liu [1], Xiaoping Wang [3], Hui Ai [1, *]

[a] These authors contributed equally to this work.

Supplementary figure legends

Figure S1. Binding curve and blue shift in fluorescence intensity of MvitPBP1-3 with fluorescence probe 1-NPN

Figure S2. Molecular docking of MvitPBP2 and sex pheromone ligands. (A) E10E12-16: Ald; (B) E10E12-16: OH; (C) E10-16: Ald; (D) E10-16: OH.

Figure S3. Molecular docking of MvitPBP3 and sex pheromone ligands. (A) E10E12-16: Ald; (B) E10E12-16: OH; (C) E10-16: Ald; (D) E10-16: OH.

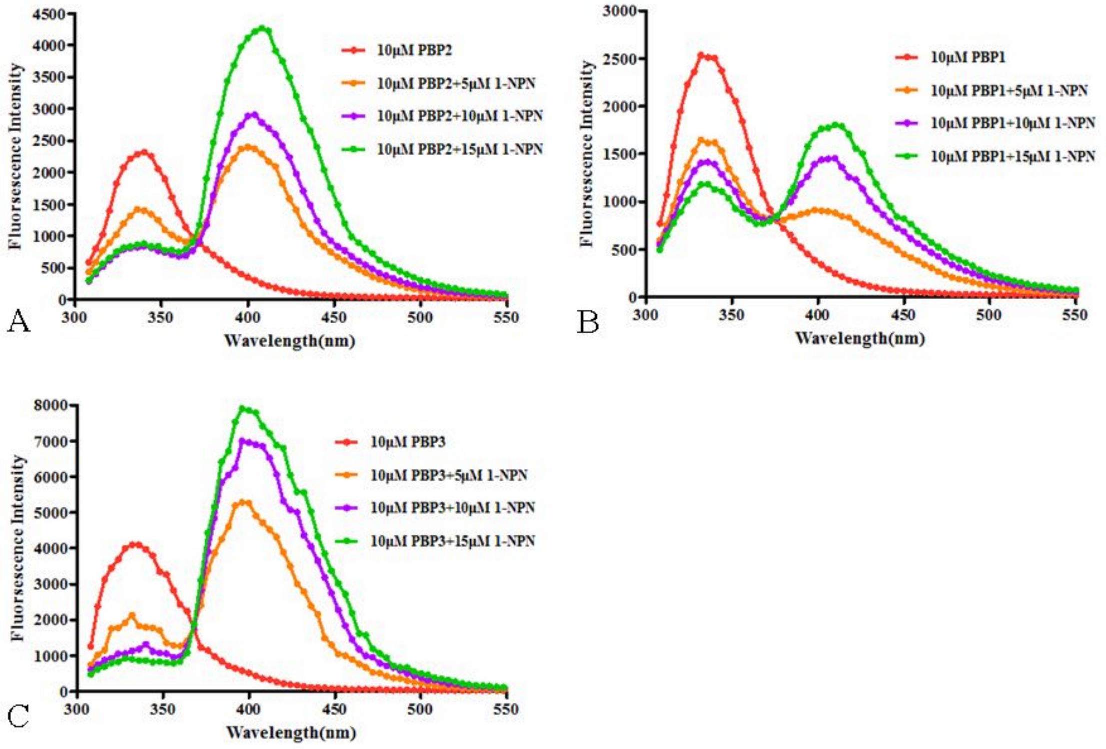
A
B
C
Fluorsescence Intensity
Wavelength(nm)
10μM PBP2
10μM PBP2+5μM 1-NPN
10μM PBP2+10μM 1-NPN
10μM PBP2+15μM 1-NPN
10μM PBP1
10μM PBP1+5μM 1-NPN
10μM PBP1+10μM 1-NPN
10μM PBP1+15μM 1-NPN
10μM PBP3
10μM PBP3+5μM 1-NPN
10μM PBP3+10μM 1-NPN
10μM PBP3+15μM 1-NPN

Figure S1

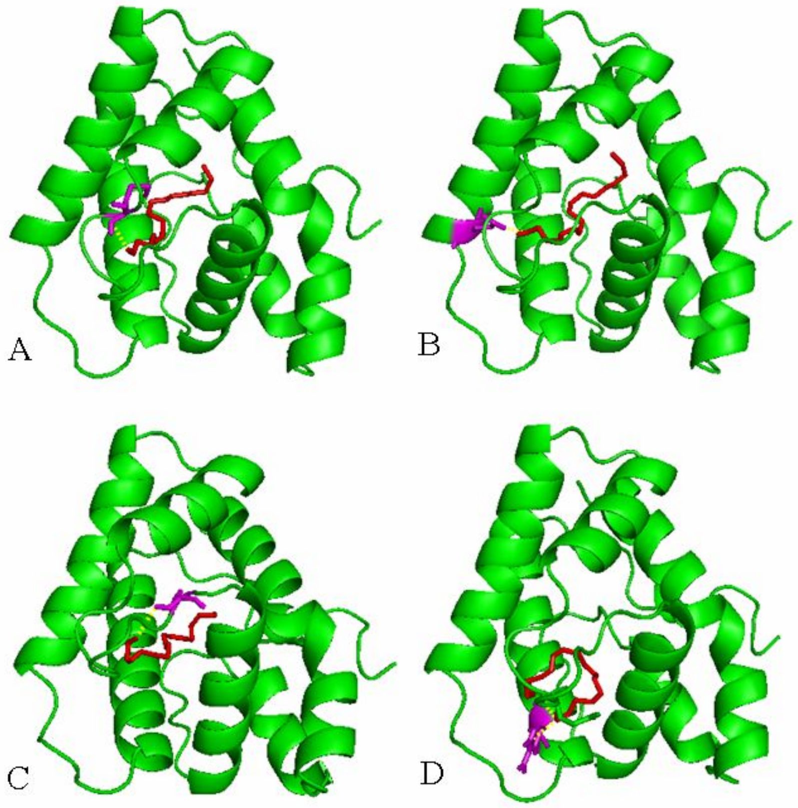

Figure S2

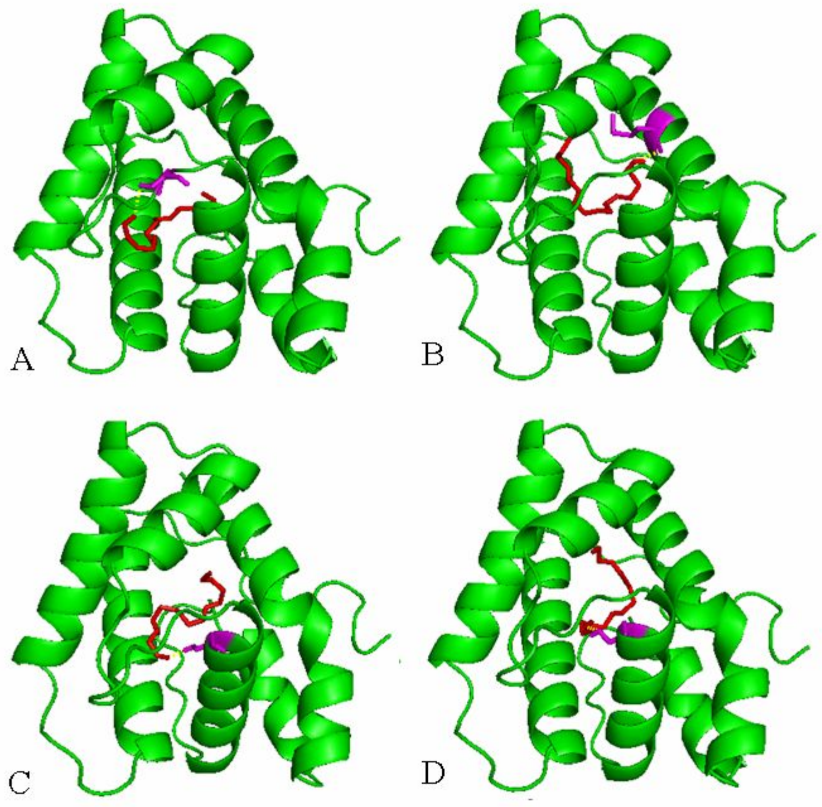

Figure S3
